# A three-dimensional topology of complex I inferred from evolutionary correlations

**DOI:** 10.1186/1472-6807-12-19

**Published:** 2012-08-03

**Authors:** Philip R Kensche, Isabel Duarte, Martijn A Huynen

**Affiliations:** 1 Center for Molecular and Biomolecular Informatics / Nijmegen Center for Molecular Life Sciences, Radboud University Medical Center, PO Box 9101, Nijmegen, HB, 6500, The Netherlands; 2 Netherlands Bioinformatics Centre, Geert Grooteplein 28, Nijmegen, GA, 6525, The Netherlands

**Keywords:** Eukaryotic complex I, Quaternary topology, Assembly, Mirror-tree method, Co-evolution

## Abstract

**Background:**

The quaternary structure of eukaryotic NADH:ubiquinone oxidoreductase (complex I), the largest complex of the oxidative phosphorylation, is still mostly unresolved. Furthermore, it is unknown where transiently bound assembly factors interact with complex I. We therefore asked whether the evolution of complex I contains information about its 3D topology and the binding positions of its assembly factors. We approached these questions by correlating the evolutionary rates of eukaryotic complex I subunits using the mirror-tree method and mapping the results into a 3D representation by multidimensional scaling.

**Results:**

More than 60% of the evolutionary correlation among the conserved seven subunits of the complex I matrix arm can be explained by the physical distance between the subunits. The three-dimensional evolutionary model of the eukaryotic conserved matrix arm has a striking similarity to the matrix arm quaternary structure in the bacterium *Thermus thermophilus* (rmsd=19 Å) and supports the previous finding that in eukaryotes the N-module is turned relative to the Q-module when compared to bacteria. By contrast, the evolutionary rates contained little information about the structure of the membrane arm. A large evolutionary model of 45 subunits and assembly factors allows to predict subunit positions and interactions (rmsd = 52.6 Å). The model supports an interaction of NDUFAF3, C8orf38 and C2orf56 during the assembly of the proximal matrix arm and the membrane arm. The model further suggests a tight relationship between the assembly factor NUBPL and NDUFA2, which both have been linked to iron-sulfur cluster assembly, as well as between NDUFA12 and its paralog, the assembly factor NDUFAF2.

**Conclusions:**

The physical distance between subunits of complex I is a major correlate of the rate of protein evolution in the complex I matrix arm and is sufficient to infer parts of the complex’s structure with high accuracy. The resulting evolutionary model predicts the positions of a number of subunits and assembly factors.

## Background

NADH:ubiquinone oxidoreductase (complex I) is with about 1000 kDa [1,2] the largest of the five complexes of the oxidative phosphorylation (OXPHOS) and a major contributor to the proton motive force that drives the ATP production by ATP-synthase [3]. Complex I has an L-shape with a hydrophilic matrix arm that protrudes into the cytoplasm in bacteria or the mitochondrial matrix in eukaryotes and a hydrophobic membrane arm. The canonical “core” of complex I consists of 14 subunits that originate from three pre-existing evolutionary modules [4]. The N-module at the distal end of the matrix arm contains flavin-mononucleotide (FMN) that accepts electrons from a donor, usually NADH. The electrons are transported through a chain of iron-sulfur (FeS) clusters along the matrix arm towards the joint of the two arms at the membrane. This membrane-proximal part of the matrix arm represents the Q-module in which the electrons are transferred to ubiquinone (Q). The energy freed by the electron-transfer is transmitted along the P-module (NADH1-6/4L) that uses the energy to pump protons across the membrane [5-7].

In diverse taxa, the canonical core of complex I has been extended by further subunits. For instance, complex I in *Thermus thermophilus* contains an additional subunit located at the interface of the N- and Q-modules [8] and a recent analysis of complex I in the α-proteobacterium *Paracoccus denitrificans* identified three additional subunits [9]. Eukaryotes obtained complex I with the endosymbiotic uptake of an α-proteobacterium that gave rise to the mitochondria. Following the endosymbiosis, the mitochondrial genome was reduced and the genes encoding matrix arm subunits of complex I were transferred to the nucleus. Additionally, complex I was extended to up to 45 subunits by so-called “accessory” or “supernumerary” subunits [1,10]. This set of permanent subunits is further extended by a number of assembly factors absent from the mature complex [11-19].

Up to now, the structures of the complete complex in the eubacterium *Thermus thermophilus*[5] and the eukaryote *Yarrowia lipolytica*[6] have been published. However, the latter structure is of a too low resolution to allow identification of the positions of the supernumerary subunits. Approximate subunit positions within the eukaryotic complex are hinted at by various types of experiments, mostly from sub-complexes observed by fractionation or as assembly intermediates (e.g. [1,20]). For instance, the application of chaotropic detergents to the bovine complex produces the three sub-complexes Iα, Iλ, and Iγ. Because these sub-complexes are large, they provide only rough information about subunit positions. For instance, Iα represents an extended Iλ sub-complex and the additional subunits could in principle be located anywhere on the surface of the Iλ sub-complex. Only limited data are available from yeast-two-hybrid [12,21], co-immunoprecipitation [14], or cross-linking [22] experiments. The identification of the positions of the assembly factors is hampered by the temporariness of the assembly intermediates and our incomplete understanding of the assembly process.

The increasing number of genome sequences allows making predictions of physical interactions by evolutionary correlation methods, including the co-occurrence of genes or phylogenetic profiling [23-26], the mirror-tree approach [27], and residue correlation [28-31], which have successfully identified new complex I subunits and assembly factors [21,25] (reviewed in [32]) and predicted relations between the five OXPHOS complexes [33]. Of these methods, residue correlation is based on the most direct evidence of physical interaction, namely the compensatory mutations at sites of interacting residues to maintain the structure of a protein or complex. By contrast, the mirror-tree method detects co-evolution more indirectly by correlating sequence similarity matrices between orthologous groups [27]. The similarities between protein sequences depend both on species divergence times and on rates of evolution. By removing the similarity due to the species divergence times [34,35], one obtains similarities that are more related to evolutionary rates. A high correlation in evolutionary rates between protein families can be evidence of a direct physical relation between proteins. For instance, if there is selection to maintain the interaction of two proteins then disrupting mutations have to be compensated for at the rate that they occur. Therefore, to maintain the interaction, an increased rate of change in one protein needs to be compensated for *at a similar rate* in the other protein. Note that the pairwise correlations in evolutionary rates between proteins can also be due to indirect interactions [36].

Here we ask whether we can use evolutionary rate correlations to predict the three-dimensional (3D) conformation of complex I. To this aim, we analyzed the evolutionary rate correlation between 38 subunits and 7 assembly factors of human complex I using the mirror-tree method [35] and find that subunits that are known to be physically close in complex I tend to show a higher degree of correlation in evolutionary rates than those that are physically distant. In the conserved core of the matrix arm, this correlation is strong enough to construct a 3D model with striking similarity to the bacterial reference structure. In a second evolutionary model that includes the 14 canonical core subunits, the membrane and matrix arms appear as clearly separated groups. Finally, we calculated a third evolutionary model including 38 subunits and seven assembly factors. This last model retains some features of the physical structure, including the separation of the matrix and membrane arms and a proximodistal axis in the matrix arm. We discuss the positions of the seven assembly factors in this model and make specific predictions about the association of some assembly factors with each other and with the permanent subunits.

## Results

### Correlation of physical and co-evolutionary distances in the conserved core

Because our study aims at complex I of human we selected 38 subunits and seven assembly factors of the human complex that have a sufficient number of orthologs for the application of the mirror-tree method (Additional file 1: Table S1). Shortly, we collected homologs of the 45 proteins by querying the nr database. All orthologous sequence sets were aligned and highly variable alignment columns were filtered out using BMGE [37]. Note that NADH3, 4L and 6 could not unambiguously be located in our reference – the structure of the complete complex I of *T. thermophilus*[5] (PDB:3M9S). However, these subunits are known to be direct neighbors and we decided to treat them as a single unit, termed NADH34L6. We calculated maximum-likelihood trees from the alignments [38] and obtained a distance matrix for each protein family from which we removed the common signal of the phylogeny [35]. The phylogeny-corrected matrices were correlated and the resulting correlation matrix was transformed into the distance matrix (see Methods). For the purpose of this article we call these distances “co-evolutionary” but note that the signal measured by the mirror-tree method is also determined by expression and general functional relatedness [39,40].

**Figure 1:**
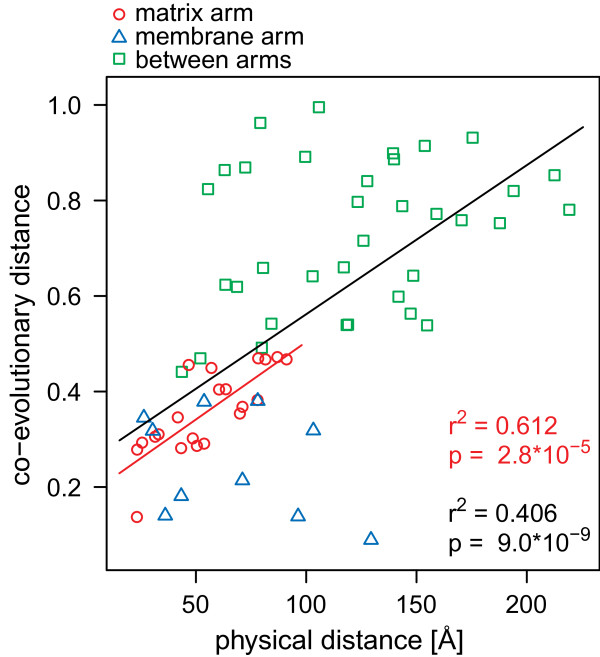
**Co-evolutionary distance correlates with physical distance.** The figure shows the distances for the subunits in the evolutionary conserved core in *T. thermophilus*[5] (PDB:3M9S). Note that in the bacterial structure NADH3, 4L and 6 were not identified individually but are neighboring. Therefore, we calculated the co-evolutionary distances with concatenated alignments of these subunits. The red line is the regression line for the matrix arm and the black line is the regression line for the complete set of points.

First we examined how well the co-evolutionary distances correspond with the distances between the 14 subunits of the conserved core of complex I (Figure 1). We compared the co-evolutionary distances to the distances of the centers of mass of the protein (see Methods). We will refer to these latter distances as “physical distances” and to the arrangement of the subunit centers in three dimensions as the “quaternary topology” of complex I. A direct comparison of the two distance measures reveals that more than 40% of the variation in the co-evolutionary distances can be explained by the physical distances of the subunit (r^2^=0.41, p=9×10^-9^, n=66; Figure 1). Within the matrix arm, physical distance explains more than 60% of the variation in co-evolutionary distances (Figure 1; r^2^=0.61, p=2.93·10^-5^, n=21). Although there is no significant correlation among the membrane arm subunits (r^2^=0.13, p=0.23, n=10), the between-arm co-evolutionary distances clearly are larger than the within-arm co-evolutionary distances, reflecting the physical separation of the two arms.

**Figure 2:**
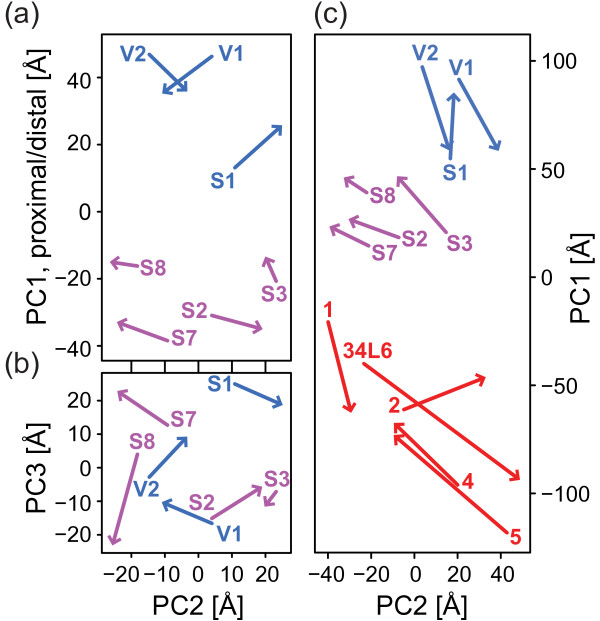
**Superimposition of the subunit centers predicted by the evolutionary model and those of the experimental model from *T. thermophilus* [**[5]**].** The arrowheads point towards the coordinates in the evolutionary configuration while the labeled arrow origins correspond to the coordinates in the experimental structure. **(a)** and **(b)** show the results for the evolutionary model of the seven conserved matrix arm subunits. The axes are the first three principal components (PC) of the evolutionary configuration. Figure **(c)** shows the results for the evolutionary model of all 14 conserved membrane and matrix arm subunits. The axes are the first two PCs derived from a PCA on the evolutionary coordinates. Blue: distal matrix arm/N-module; purple: proximal matrix arm/Q-module; red: membrane arm/P-module. Subunit names were abbreviated by omitting the “NDUF” or “NADH” prefixes.

The high correlation of co-evolutionary and physical distance suggests that it may be possible to obtain an accurate 3D model of the protein complex from the pairwise co-evolutionary distances. We used classical multidimensional scaling (cMDS) to integrate the co-evolutionary distances between the seven matrix arm core subunits into an evolutionary 3D configuration. To ensure that the 3D configuration reliably reflects the co-evolutionary distances, we calculated the *P*_*3*_-value of the configuration, a cMDS-specific goodness-of-fit measure that is analogous to the fraction of variation explained by the first three eigenvalues in a principal component analysis [41] (see Methods). The *P*_*3*_-value of the evolutionary configuration is 0.89 and thus close to that of a perfect fit (1.0) and well above the cutoff 0.8 suggested as desirable [41]. Next, we compared the evolutionary 3D model with the bacterial structure (Figure 2a; see Methods). The correlation of the distances in the 3D configuration with the distances in the bacterial structure is lower (r^2^=0.55) than that of the raw co-evolutionary distances (r^2^=0.61) but still significant (p=1.28·10^-4^, n=21). The root mean square deviation (rmsd) of the bacterial quaternary topology with the eukaryotic evolutionary configuration is 18.7 Å, which compares well to the about 180 Å length of the matrix arm [8]. We subjected the evolutionary configuration to a principal component analysis and used the principal components thus obtained as new coordinate system for the configuration (Figure 2a/b). The proximodistal axis of the matrix arm core corresponds to the first and largest principal component (Figure 2a) and is thus correctly identified by the evolutionary model as the axis with the largest extent. The positioning of subunits along this axis is almost perfectly recovered by the evolutionary model (correlation bacterial/predicted along the first axis: r^2^=0.95, p=2.1×10^-4^, n=7). Also the second axis shows a strong correlation (r^2^=0.62, p=3.5×10^-2^) while the correlation along the third axis (r^2^=0.45) is significant at a level of 9.8% (p=9.8×10^-2^). The projection of the second and third axes (Figure 2b) shows that among both the proximal Q-module subunits and distal N-module subunits the evolutionary model identifies the correct circular ordering around the proximodistal axis. Furthermore, in the predicted model, which is based on eukaryotic sequences only, the four Q-module subunits (Figure 2b; purple) are twisted relative to the three N-module subunits (Figure 2b; blue) compared to the bacterial structure. Interestingly, a twist in the same direction was observed in a comparison of the matrix arms of the eukaryote *Y. lipolytica* and *T. thermophilus* ([6], personal communication): When looking from the matrix towards the membrane the N-module is turned clockwise relative to the Q-module.

After the prediction of the topology of the seven matrix arm core subunits, we predicted the topology of the complete core of 14 matrix- and membrane arm subunits that are conserved among bacteria and eukaryotes. Again, the co-evolutionary distances of the subunits can be well embedded in three dimensions (*P*_*3*_=0.84) and result in a configuration with separate membrane and matrix arms (Figure 2c). The rmsd of this evolutionary model and bacterial structure is 47.6 Å, which corresponds to about 25% of the length of the membrane arm [5]. Also in the complete-core model, the proximodistal axis of the matrix arm is recovered (r^2^=0.96, p=9.3×10^-5^) although the rmsd of the matrix arm core subunits to the bacterial structure is lower (34.0 Å) than that for the configuration that only contained matrix arm subunits (18.7 Å). As expected from the correlation between the pairwise physical distances and their evolutionary correlation, the accuracy of the positioning in the membrane arm is poor (rmsd=61.8 Å). A closer inspection of the evolutionary model suggests that the membrane arm subunits are located as a cluster more sideways of the matrix arm than in the physical structure. In the superimposition of the two structures, this manifests in a tendency of the Q-module away from the membrane and a slight tendency of the distal N-module towards the membrane.

### Eukaryotic complex I and assembly factors

**Figure 3:**
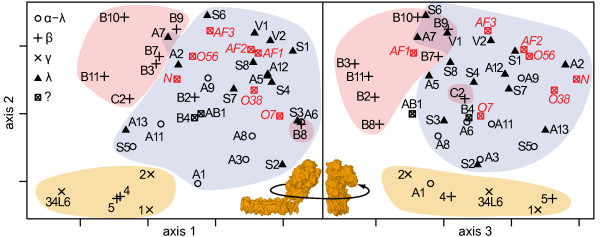
**Evolutionary model of 38 subunits and seven assembly factors of human complex I.** Only the predicted subunit centers are displayed. The evolutionary model was first rotated and scaled to fit to the bacterial structure and both models were then manually rotated into the orientations of the bacterial complex shown in the insets. The symbols code the sub-complex association according to Vogel et al. [93]. Sub-complex Iα-λ represents the fraction of large sub-complex Iα that is not also contained in the smaller matrix arm sub-complex Iλ. Category “?” represents subunits that have not been assigned to any sub-complex. Assembly factors are labeled in italics and red. Subunit names were abbreviated by omitting the “NDUF” or “NADH” prefixes where applicable. N: NUBPL; O7: C20orf7; O38: C8orf38; O56: C2orf56. An interactive three-dimensional model of the evolutionary configuration is provided in Additional file 2.

Although the structure of the 14 core subunits in bacteria is known [5] and largely conserved in eukaryotes [6], the arrangement of the mostly eukaryote-specific accessory subunits has not yet been resolved. Furthermore, the transient nature of the interaction of assembly factors in complex I assembly intermediates hampers the identification of their binding sites in the complex. We therefore asked whether the positions of the accessory subunits and assembly factors could be identified from their evolutionary correlation. We extended the evolutionary 3D configuration to include 38 permanent subunits and 7 assembly factors of the human complex. Both the goodness-of-fit of the 3D configuration with the raw co-evolutionary distances and the comparison with the reference structure, indicate the quality of this model. The goodness-of-fit measure *P*_*3*_, which expresses how well the 3D model represents the pairwise co-evolutionary distances, is lower (*P*_*3*_=0.43) than for the previous models. However, a comparison of this *P*_*3*_-value with the distribution of *P*_*3*_-values of 10^6^ permuted symmetric matrices fitted in 3D (*P*_*3*_^random^=0.17±0.005) shows that the arrangement of the distances in the matrix fits significantly better in a 3D configuration than random arrangements (p<10^-6^). The co-evolutionary distances between the 45 proteins are thus highly consistent with a 3D representation. Furthermore, the 3D configuration captures 72% of the variation in the co-evolutionary distances (r^2^=0.72; raw distances versus embedded distances). In the comparison with the bacterial reference structure, the model of 45 proteins has a slightly lower rmsd (52.6 Å) than the model of only 14 conserved subunits. The extended model clearly recapitulates a number of known elements of the physical structure of the complex (Figure 3). Axis 1 in Figure 3a separates the membrane arm (left) and matrix arm (right) subunits. Among the membrane arm subunits of the Iβ and Iγ sub-complexes (3a, left), axis 2 differentiates between subunits that tend to be encoded by the mitochondrial genome (bottom) and nuclear-encoded (top) subunits. The two subunits NDUFB8 (3a, bottom/right) and NDUFC2 (3b, middle) are located somewhat separate from the remaining Iβ subunits. Among the matrix arm subunits, axis 2 differentiates between the distal N-module subunits V1, V2, and S1 (3a right/top) and the proximal Q-module subunits S7 and S8 (middle), S2, and S3 (bottom). The accessory subunits of hydrophilic Iλ matrix arm sub-complex (Figure 3, λ) show a tendency towards the top, while those of the Iα sub-complex that are not part of Iλ (3a, α-λ) tend towards the membrane arm subunits at the bottom. The association of this latter group of subunits with the proximal matrix and membrane arm is strongly supported by experimental data (S5 [42,43], A9 [20,44-46], A3 [1,2,47], A6 [48], A8 [48,49], A11 [50], A1 [21]). All assembly factors are located close to the matrix arm subunits. NDUFAF2 (AF2), NDUFAF3 (C3orf60, AF3), C8orf38 (O38), C2orf56 (O56), and NUBPL (N) as well as the permanent subunits A2 and A9 sit on one side of the distal matrix arm core subunits V1, V2, and S1. Only C20orf7 (O7) is placed close to the proximal matrix arm subunits S2, S3 (Figure 3b, right bottom) and the proximal membrane arm subunits A3, A6, and A8. Assembly factor 1 (AF1) is positioned close to the Iλ subunits S8 and A5.

## Discussion

Our results show that the evolutionary rates of complex I subunits contain a significant amount of information about the complex’s quaternary structure. For the matrix arm we found that about 61% of the correlation in evolutionary rate could be explained by the distances of the subunit centers. This is even more striking if we consider that the evolutionary model was derived from eukaryotic sequences and thus should reflect the matrix arm structure in eukaryotes, while our reference structure is from a bacterium. Indeed, the evolutionary 3D model revealed a twist between the N-module and the Q-module when compared to the bacterial structure, a finding that is supported by experimental data ([6], personal communication).

In the two models that include the membrane arm, mitochondria-encoded subunits were predicted to be separate from nucleus-encoded subunits, which is in line with previous results. The independent variation of evolutionary rates in the nuclear and mitochondrial genomes [51] may have contributed to the isolation of the mitochondria encoded subunits in our models. Nevertheless, we stress that the position of the membrane arm core subunits, specifically at the proximal end of the matrix arm in both models, indicates a signal of the physical structure in the evolutionary correlation data. Furthermore, the strict separation of the nucleus-encoded Iβ subunits and their mitochondria-encoded counterparts NADH4 and 5 may be explained by other factors, as these two groups also behave differently in experiments [1,52,53]. Interestingly, despite its nuclear encoding, the membrane-integral subunit NDUFA1 of the Iα sub-complex [1,2,54] is positioned close to the membrane arm core, in particular close to NADH2 (Figures 3a and 3b). In *T. thermophilus*, NADH2 is located between the two subunits NADH1 and NADH4 [5] both of which are known physical interactors of NDUFA1 [21]. A direct physical interaction of NDUFA1 with NADH2 is therefore likely.

The evolutionary correlation failed to identify the correct topology of the membrane arm core. A number of biological reasons could explain such a lack of signal. First, long-range structural constraints [5,6] may interfere with the distance-dependent structural constraints that are necessary for a distance-dependent strength of evolutionary rate correlation. Second, the formation of OXPHOS super-complexes with complex I dimers may result in correlations between distant subunits. Indeed, despite their positions at opposite ends of the membrane arm, NADH1 and 5 show a high correlation in evolutionary rates with each other and with subunit CYTb of complex III [33] consistent with their proximity in OXPHOS complexes organized into respiratory strings [55]. Third, the lack of correlation with physical distance may result from non-adaptive variation in the mitochondria-encoded genes caused by variable and, at least in some eukaryotic taxa, heterogeneous mutation-pressure [56]. Indeed, in a number of animal taxa changes in gene order or mutation-pressure led to non-adaptive changes in mitochondrial genes [57-59]. The mitochondrial genomes of some taxa in our study, such as plants, are clearly different from those in animals (reviewed in [60]) and their genes are likely under different mutation-pressures [61]. Fourth, the embedding of the membrane proteins in two dimensions might reduce the evolutionary constraints to maintain interactions in comparison to proteins that are embedded in three dimensions.

The integration of multiple proteins in a single model assumes that the interactions are permanent and non-competitive. This is clearly not the case for the model of 45 proteins because it includes assembly factors. This model can therefore not exactly represent a physical structure. According to current models, complex I assembles from independent subcomplexes [62]. Of the assembly factors required for this process and included in our study, only NDUFAF1 (AF1) is required for the assembly of the distal membrane arm sub-complex [13,63,64]. In our model, AF1 is located close to the matrix arm, which supports an indirect rather than a direct involvement of AF1 in membrane arm assembly [65]. The distal membrane arm further combines with a pre-formed membrane-anchored proximal matrix/membrane arm that contains the subunits NDUFS2 (S2) and NADH1 (1) and possibly NDUFS3 (S3) and NDUFS7 (S7) [62,64] and whose assembly involves NDUFAF3 (AF3) and possibly C8orf38 (O38) [17,66]. Although the membrane-association of AF3 and O38 is not reflected in our data, they form a tightly co-evolving triple with C2orf56 (O56), which is known to bind the proximal matrix arm subunit S2 [12]. The high correlation in evolutionary rates between AF3, O38, and O56 suggest strong selective constraints on their cooperation during the assembly of the proximal matrix/membrane arm sub-complex. The fourth assembly factor that has been experimentally linked to the proximal membrane arm, C20orf7 (O7) [18,64], is indeed placed close to the proximal matrix arm subunits S2, S3 (Figure 3b, right bottom), and the proximal membrane arm subunits A3, A6, and A8 [2,47,48].

After the joining of the two membrane arm intermediates, the proximal matrix arm is further extended. This step involves the NUBPL-mediated assembly of at least one FeS-cluster into the distal matrix arm [11,67]. In the evolutionary configuration the assembly factor NUBPL is positioned side by side with the permanent subunit NDUFA2 (A2; Figure 3b, right top). Like NUBPL, A2 is associated with the distal matrix arm [68]. The highly conserved A2 subunit is structurally similar to thioredoxin-like proteins with a loop-region of probably variable conformation that contains two cysteines in human (C24 and C58) [69]. These cysteines can form a revertible disulfide bridge with an *in-vitro* redox-potential in the range of the large majority of isopotential FeS-clusters of complex I [69,70]. Although the cysteines are not fully conserved, occasionally FeS-clusters are bound by serine, histidine, or aspartate [71]. Indeed, the human serine 30 in NDUFA2 is a good candidate for FeS-cluster binding because it is perfectly conserved in all species, with the notable exceptions of *Trypanosoma* and *Leishmania*, in which it is substituted by cysteine. Together these observations and the very strong evolutionary rate correlation of A2 and NUBPL support an involvement of A2 in complex I associated FeS-cluster assembly or maintenance. The peripheral position of A2 and NUBPL in the model could be a consequence of other strong evolutionary constraints not directly related to complex I.

Also NDUFAF2 (AF2, B17.2L) has been linked to the assembly of the distal matrix arm [14,64]. Interestingly, the evolutionary data position AF2 directly besides its paralog NDUFA12 (A12, B17.2) [10,14]. Like AF2, A12 is known to be associated to the distal-matrix arm to which it is directly recruited from the mitochondrial matrix [68]. The correlation in evolutionary rates and the independent co-loss in multiple complex I lacking taxa [10] support an evolutionarily conserved functional relationship of AF2 and A12. It is tempting to speculate that AF2 temporarily binds at the binding site of A12, e.g. to stabilize the local structural context, and is later substituted by its paralog. Such close positioning and physical interaction of homologous proteins within the same protein complex is one of the prevailing trends in the “fate” of duplicated proteins in complexes [72]. Complex I appears to add another twist to this pattern in the sense that the predicted interaction is only temporary.

The rate of protein evolution is influenced by diverse factors [73], in particular expression and general functional relatedness [39,40,74]. It is therefore even more remarkable that we found physical distance to be the major determinant of the evolutionary rate correlation for the complex I matrix arm. However, this result does not apply to the whole complex. Thus, to establish whether the mirror-tree/MDS combination is a good general method to predict quaternary structures, other complexes need to be analyzed. Furthermore, instead of using the mirror-tree method one could use residue correlation to measure the co-evolution of subunits more directly. Residue correlation has been used to predict contact interfaces for protein pairs [30,75] and to investigate a rotation-symmetric homo-multimeric complex [76]. A simple implementation would be to integrate pairwise residue correlations [28] or correlations that account for indirect correlations [30,31,77] or phylogenetic dependency [76,78] by in-silico two-hybrid [80] into subunit distances and map these into three dimensions by multidimensional scaling.

## Conclusions

The correlations of evolutionary rates between subunits of the eukaryotic complex I contain detailed information about the structural arrangement of the matrix arm subunits. This allowed us to make specific predictions about the positions of supernumerary subunits and assembly factors of the matrix arm, which may guide further experimental investigations. Multidimensional scaling could not reconstruct the structure of the membrane arm core. A future analysis will have to investigate what may cause this lack a spatial signal along the membrane arm and thus clarify in particular the relevance of conformational dynamics and super-complex arrangement into a respiratory string for the sequence evolution of complex I.

## Methods

### Alignments

We included 38 permanent subunits and seven assembly factors of human complex I for which a sufficient number of sequences were available. We collected homologous sequences from the nr database [80] using PSI-BLAST (default parameters). Multiple queries from different species were used whenever PSI-BLAST failed to find known homologs [see Additional file 1. For A6, B9, A12, and AF2, orthologous groups were manually identified in neighbor-joining trees constructed with identity matrices and correcting for multiple substitutions. Species overlap between the partitions was used to divide the trees into separate orthologous groups. The remaining subunits were treated by a different protocol. First, to ensure a separation of the paralogs NADH2, 4, and 5, we built a set of trusted orthologs of NADH2, 4, and 5 from those sequences that had the best bi-directional hit with the human query using PSI-BLAST. From these seed sequence sets we computed three HMM profiles and sorted the remaining homologs into the orthologous group to which they showed the best profile-alignment [81]. For all sequence sets we selected as single ortholog per species the sequence with the highest NEEDLE score in a pairwise alignment to the human query [82] (default parameters) and/or manual selection based on multiple alignments (MAFFT [83], CLUSTALW [84,85], HMMER [86], HHSEARCH [81]). The kinetoplastida were excluded from our analysis due to their high level of sequence divergence. To gain high quality alignments, we aligned all sequence sets with CLUSTALW and manually fixed misalignments. The manually curated alignments are provided in Additional file 3. Next, we filtered alignment columns with BMGE [37] (−m BLOSUM30 -g 0.50 -b 4), removed sequences that had more than 33% gaps, and restricted the alignments to those species for which we found at least eight subunits of the complex. Of the 43 alignments, 39 had more than 75 sequences and there was no alignment with less than 44 sequences. Finally, we calculated phylogenetic trees using RAXML [38] (Version 7.2.6, PROTGAMMAMTREV for NADH1/2/3/4/4L/5/6, otherwise PROTGAMMAJTT; 4 rate categories) [see Additional file 4. A single tree was calculated for the concatenated alignment of NADH3, 4L, and 6.

### Evolutionary correlation

We calculated evolutionary correlation using a variant of the mirror-tree method [27]. Every subunit’s tree was transformed into a vector *v* containing the pairwise distances between pairs of species in the tree. Because all subunit’s trees represent the evolution of proteins within the same species phylogeny they all are similar to that phylogeny and to each other. To remove this basic similarity of the distances we applied the orthogonal projection method developed by Sato *et al.*[35]. The method projects each sequence distance vector *v* on a reference distance vector *p* that represents the underlying species phylogeny. Let *v*_*p*_ be the projection of *v* onto *p,* then the corrected vector v* is the residual vector *v - v*_*p*_. The corrected sequence distance vector is thus calculated by

(1) $$v^{*}=v-v_{p}=v-\frac{p^{T}v}{p^{T}p}p$$

with the row vector *p*^*T*^[87]. We derived the reference vector *p* directly from the subunits’ distance vectors, as suggested by Kann *et al.*[88]. Specifically, the reference distance between a pair of species was calculated as the average of the distances between these species in the trees of the complex I subunits. Note that this choice of reference as an average of the analyzed vectors also removes the specific pattern of co-variation in evolutionary rates that reflects selective pressure on the complex as a whole. It thus focuses the results on distances between the subunits rather than their distances to unrelated proteins. Finally, the corrected distance vectors were correlated with each other by Spearman rank correlation to yield the subunits' co-evolutionary similarity [27]. We required that the species pairs were present in at least five of our 43 distance vectors. Species pairs occurring in fewer than five vectors were ignored in the correlations. Note that our choice of the set of subunits included the requirement that there are at least 15 species in all pairs of alignments. Only for 17 out of 903 subunit pairs, the correlation values were based on less than 30 species. The mirror-tree method has the advantages of being easily implemented and it requires low computational resources, even with correction for the basic correlation due to the shared phylogeny.

### Multidimensional scaling (cMDS)

The co-evolutionary similarities *r* were linearly transformed into dissimilarities *d* by first taking the inverse with respect to the maximum correlation coefficient, i.e. *d'*=1-*r*, and then rescaling to the interval [0,1] using *d* = *d'* / max(*d'*) [see Additional file 5. This transformation considers negative correlations in evolutionary rates as negative evidence of physical interaction. We used classical multidimensional scaling (cMDS) as implemented by the R function cmdscale [89] (default parameters) to find the matrix ** *X* ** of coordinates of *n* points (rows, subunits) in *n* dimensions (columns) such that the distances between the embedded points are as similar as possible to the original co-evolutionary dissimilarities. Our description of cMDS closely follows that by Borg and Groenen [90]. In detail, for a dissimilarity matrix **Δ**, cMDS minimizes the loss function L(X) = ||** *XX* **^T^*–*** *B* **_**Δ**_||^2^, where ** *XX* **^T^ is the scalar product matrix of the embedded coordinates and **B**_Δ_ = − 1/2 **JΔ**^(2)^**J** is the double centered squared dissimilarity matrix with the centering matrix **J** = **I** - n^-1^**11**^T^**I** is the identity matrix, and **1** is a *n* x 1 matrix of 1s. The solution is found analytically by eigen-decomposition of **B**_**Δ**_**= QΛQ** and calculation of **X** = ** *Q* **_*+*_** *Λ* **_*+*_^1/2^, where ** *Λ* **_*+*_ represents the matrix of the largest *k* eigenvalues greater than zero and ** *Q* **_*+*_ the corresponding columns of ** *Q* **. The relative magnitudes of the eigenvalues in ** *Λ* ** correspond to the relative contributions of the columns of ** *X* ** in explaining the raw dissimilarities. The goodness-of-fit of the cMDS configuration of *n* subunits in the *k* dimensions is quantified by *P*_*k*_ (formula 5.2 in [41]):

(2) $$P_{k}=\frac{\sum_{i=1}^{k}\lambda_{i}}{\sum_{i=1}^{n-1}\lambda_{i}}$$

where *λ*_*i*_ is the *i*-th largest eigenvalue of ** *B* **_**Δ**_. Note that the relation between the co-evolutionary dissimilarity and the distance in the cMDS configuration (Shepard diagram, Additional file 1) indicates that the 3D configuration reflects the raw co-evolutionary distances over its whole range.

### Superimposition of configurations

The structure of complex I in the *T. thermophilus* served as our reference [5] (PDB:3M9S). We approximated the mass centers of the subunits as the average of x, y, and z coordinates of their C_β_ atoms (C_α_ for glycine) [91]. The evolutionary configuration was fitted by rotation and isometric scaling on the bacterial configuration using generalized Procrustes analysis as implemented in the function GPA of the R package FactoMineR (Version 1.14) [92]. We quantified the difference between the bacterial configuration *T* and the evolutionary configuration *C* of *n* subunits by their root mean square deviation (rmsd)

(3) $$rmsd(T,C)=\sqrt[{2}]{\frac{\sum_{i=1}^{n}|t_{i}-c_{i}{|}^{2}}{n}}$$

where |*t*_*i*_ - *c*_*i*_| is the distance between the bacterial and predicted center of the *i-*th subunit.

## Competing interests

The authors declare that they have no competing interests.

## Authors’ contributions

PRK and MAH conceived the study. PRK, ID, and MAH manually curated the alignments. PRK analyzed the data and wrote the article. All authors read and approved the final manuscript.

## Supplementary Material

Additional file 1 **Microsoft Word 97 Document.** Table of human complex I members and query sequence identifiers and Shepard diagrams for the three discussed models [1,17][94-100]. Click here for file

Additional file 2 **Zip-compressed FASTA alignment files.** Manually curated and unfiltered alignments. The FASTA header lines contain (1) a short sequence identifier consisting of a number and the abbreviated species name and (2) a long sequence identifier with the number written between the genus and epithet of the species name. Sequence gaps are indicated by '-'. Subunits NADH3, NADH4L, and NADH6 were combined ("nadh34L6"). Click here for file

Additional file 3 **Zip-compressed New Hampshire eXtended (NHX) tree files.** Gene trees. Click here for file

Additional file 4 **Tabulator delimited text file.** Matrix of pairwise co-evolutionary distances. Click here for file

Additional file 5 **VRML97 format.** Interactive visualization of the evolutionary configuration. The predicted subunit centers are labeled by the abbreviations used in the article and color-coded according to sub-complex membership [see Additional file 1]. Specifically, most subunits are abbreviated by omitting the "NADH" or "NDUF" prefix, with the exception of NUBPL, C20orf7, C8orf38, and C2orf56 that are abbreviated to N, O7, O38, and O56, respectively. The sub-complexes are Iλ (blue), Iα-λ (white), Iγ (yellow), and Iβ (red). Subunits without sub-complex association are shown in purple. You can display the VRML97 file of the configuration using a VRML viewer like Flux Player (Windows; http://mediamachines.wordpress.com/flux-player-and-flux-studio/) or freewrl (Windows, Linux, Mac; http://freewrl.sourceforge.net/). Click here for file
