# Supplementary material for: A three-dimensional topology of complex I inferred from evolutionary correlations
Source: BMC Struct Biol. 2012 Aug 3;12:19. doi: 10.1186/1472-6807-12-19 (PMC3436739; doi:10.1186/1472-6807-12-19)
Supplement: Additional file 1 — Microsoft Word 97 Document. Table of human complex I members and query sequence identifiers and Shepard diagrams for the three discussed models [1,17][94-100]. [file 1472-6807-12-19-S1.doc]

# Additional File 1

# Co-evolution predicts the three-dimensional topology of complex I

Philip R. Kensche, Isabel Duarte, Martijn A. Huynen

**Table.** The subunits and assembly factors of complex I that were included in the analysis. The assembly factors are highlighted in bold. The columns are the abbreviation used in the text, the subunit name in the bacterium *Thermus thermophiles* and in human, the sequence identifier of the query sequence(s) used for the data collection, the phylogenetic origin of the subunit and information about the approximate positions of the subunits.

| **Abbreviation** | **Thermus** | **Human** | **Queries** | **Origin** | **Subcomplex/Comments** |
| --- | --- | --- | --- | --- | --- |
| 1 | Nqo8 | NADH1 | NP_536843.1 | bacteria | γ |
| 2 | Nqo14 | NADH2 | NP_536844.1 | bacteria | γ |
| 34L6 | Nqo7 | NADH3 | NP_536850.1 | bacteria | γ |
| 4 | Nqo13 | NADH4 | NP_536852.1 | bacteria | β ; βL |
| 34L6 | Nqo11 | NADH4L | NP_536851.1 | bacteria | γ |
| 5 | Nqo12 | NADH5 | NP_536853.1 | bacteria | β ; βL |
| 34L6 | Nqo10 | NADH6 | NP_536854.1 | bacteria | α-λ |
| V1 | Nqo1 | NDUFV1 | NP_009034.2 | bacteria | λ |
| V2 | Nqo2 | NDUFV2 | NP_066552.1 | bacteria | λ |
| S1 | Nqo3 | NDUFS1 | EAW70379.1 | bacteria | λ |
| S2 | Nqo4 | NDUFS2 | NP_004541.1 | bacteria | λ |
| S3 | Nqo5 | NDUFS3 | NP_004542.1 | bacteria | λ |
| S7 | Nqo6 | NDUFS7 | NP_077718.3 | bacteria | λ |
| S8 | Nqo9 | NDUFS8 | NP_002487.1 | bacteria | λ |
| S4 |  | NDUFS4 | NP_002486.1 | bacteria | λ ; |
| S6 |  | NDUFS6 | NP_004544.1 | bacteria | λ ; |
| A12 |  | NDUFA12 | NP_061326.1 | bacteria | λ |
| A1 |  | NDUFA1 | NP_004532.1 | eukaryota | α-λ ; membrane ; Y2H interaction with NADH1 and NADH4 |
| A11 |  | NDUFA11 | NP_783313.1, At2g42210 | eukaryota | α-λ ; hydrophobic ; Neurospora crassa mutant fails to assemble membrane arm and probably proximal matrix arm |
| A13 |  | NDUFA13 | AAG44670.1 | eukaryota | λ |
| A2 |  | NDUFA2 | NP_002479.1 | eukaryota | λ |
| A3 |  | NDUFA3 | NP_004533.1, P42117 | fungi/metazoa | α-λ |
| A5 |  | NDUFA5 | NP_004991.1 | eukaryota | λ ; Y2H interaction with B1 |
| A6 |  | NDUFA6 | NP_002481.2 | eukaryota | α-λ ; λ |
| A7 |  | NDUFA7 | CAG47062.1, P19968 | fungi/metazoa | λ |
| A8 |  | NDUFA8 | NP_055037.1 | eukaryota | α-λ |
| A9 |  | NDUFA9 | EAW88838.1 | eukaryota | α-λ |
| AB1 |  | NDUFAB1 | NP_004994.1 | eukaryota | α-λ + β ; β ; βS |
| B10 |  | NDUFB10 | NP_004539.1 | eukaryota | β ; βS/L |
| B11 |  | NDUFB11 | AAL32064.1, At3g57785, XP_960286.1 | eukaryota | β ; γ |
| B2 |  | NDUFB2 | NP_004537.1, NP_565128.1 | eukaryota | β ; βS |
| B3 |  | NDUFB3 | NP_002482.1, At1g14450 | eukaryota | β |
| B4 |  | NDUFB4 | NP_004538.2, AAM61161.1, XP_002143105.1 | eukaryota | α-λ + β |
| B7 |  | NDUFB7 | NP_004137.2 | eukaryota | β ; βS |
| B8 |  | NDUFB8 | NP_004995.1, At5g47570 | eukaryota | β ; βS |
| B9 |  | NDUFB9 | NP_004996.1 | eukaryota | β ; βS |
| C2 |  | NDUFC2 | NP_004540.1, NCU01467, At4g20150 | eukaryota | β ; membrane |
| S5 |  | NDUFS5 | NP_004543.1 | eukaryota | α-λ |
| **AF1** |  | NDUFAF1 | NP_057097.2 | eukaryota | interacts with ACAD9 and ECSIT |
| **O38** |  | C8ORF38 | NP_689629.2 | eukaryota | see discussion |
| **AF2** |  | NDUFAF2 | NP_777549.1 | eukaryota | see discussion |
| **N** |  | NUBPL | NP_079428.2 | eukaryota | see discussion |
| **O56** |  | C2ORF56 | NP_653337.1 | eukaryota | see discussion; interaction with S2 ; cooccurrence with λ subunits |
| **O7** |  | C20ORF7 | NP_077025.2 | eukaryota | see discussion |
| **AF3** |  | C3ORF60 | NP_951032.1 | eukaryota | membrane ; gene order conservation with O38 ; gene order conservation with Oxa1 ; interacts with C6ORF66 |
|  |  |  |  |  |  |


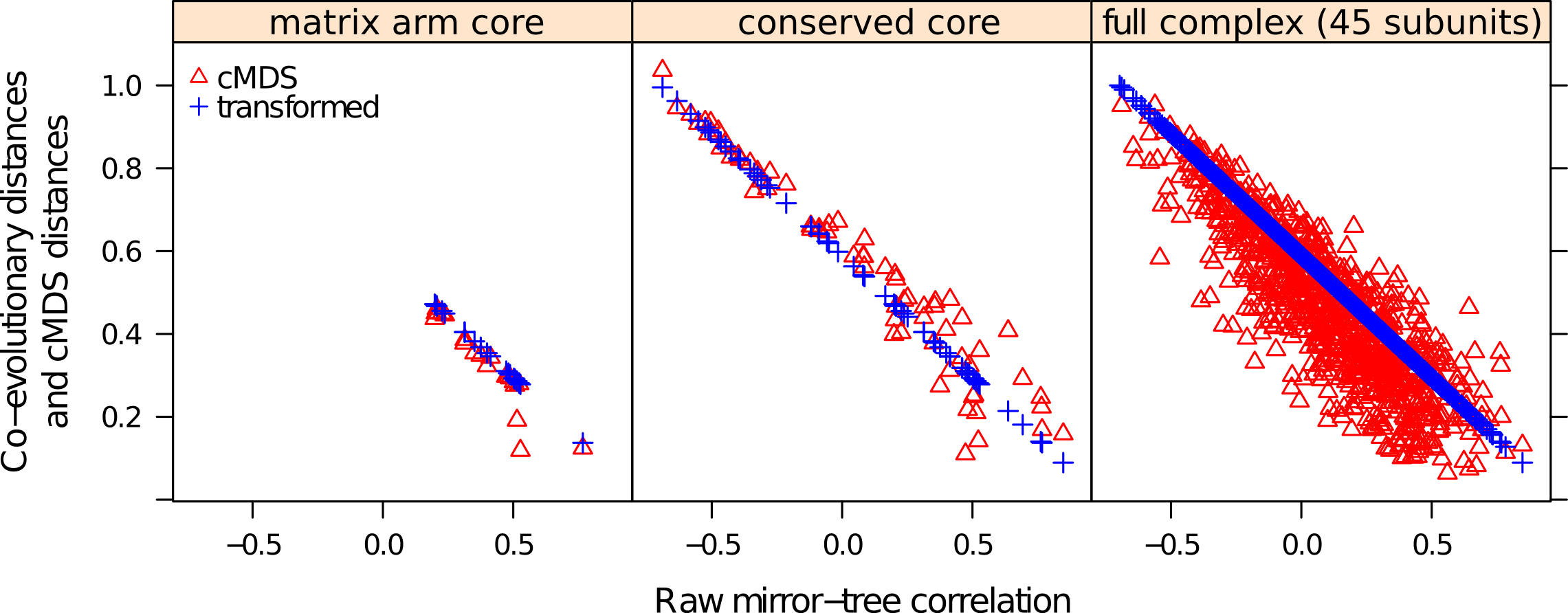


Figure S1. Shepard diagrams for all three models of the main article. The Shepard diagrams show the relation between the raw mirror-tree correlation score (x-axis), the co-evolutionary dissimilarity resulting from the (linear) transformation of the raw correlations (y-axis, blue crosses), and the distances in the cMDS configurations (y-axis, red triangles). Note that the negative raw correlations are due to the orthogonal projection. All but a single uncorrected correlations are positive (uncorrected correlations = 0.70 ± 0.15; single exception has an uncorrected correlation score of -0.056).

1. Carroll J, Fearnley IM, Shannon RJ, Hirst J, Walker JE: **Analysis of the subunit composition of complex I from bovine heart mitochondria**. *Mol Cell Proteomics* 2003, **2**(2):117-126.

2. Sazanov LA, Peak-Chew SY, Fearnley IM, Walker JE: **Resolution of the membrane domain of bovine complex I into subcomplexes: implications for the structural organization of the enzyme**. *Biochemistry* 2000, **39**(24):7229-7235.

3. Yip C-y, Harbour ME, Jayawardena K, Fearnley IM, Sazanov LA: **Evolution of respiratory complex I: 'supernumerary' subunits are present in the α-proteobacterial enzyme**. *J Biol Chem* 2010, **286**(7):5023-5033.

4. Yamaguchi M, Hatefi Y: **Mitochondrial NADH:ubiquinone oxidoreductase (complex I): proximity of the subunits of the flavoprotein and the iron-sulfur protein subcomplexes**. *Biochemistry* 1993, **32**(8):1935-1939.

5. Hirst J, Carroll J, Fearnley IM, Shannon RJ, Walker JE: **The nuclear encoded subunits of complex I from bovine heart mitochondria**. *Biochim Biophys Acta* 2003, **1604**(3):135-150.

6. Marques I, Duarte M, Videira A: **The 9.8 kDa Subunit of Complex I, Related to Bacterial Na+-translocating NADH Dehydrogenases, is Required for Enzyme Assembly and Function in Neurospora crassa**. *J Mol Biol* 2003, **329**(2):283-290.

7. Gershoni M, Fuchs A, Shani N, Fridman Y, Corral-Debrinski M, Aharoni A, Frishman D, Mishmar D: **Co-evolution predicts direct interactions between mtDNA and nuclear DNA-encoded subunits of oxidative phosphorylation complex I**. *J Mol Biol* 2010:158-171.

8. Carroll J, Shannon RJ, Fearnley IM, Walker JE, Hirst J: **Definition of the nuclear encoded protein composition of bovine heart mitochondrial complex I. Identification of two new subunits**. *J Biol Chem* 2002, **277**(52):50311--50317.

9. Nehls U, Friedrich T, Schmiede A, Ohnishi T, Weiss H: **Characterization of assembly intermediates of NADH:ubiquinone oxidoreductase (complex I) accumulated in Neurospora mitochondria by gene disruption**. *J Mol Biol* 1992, **227**(4):1032-1042.

10. Fearnley IM, Carroll J, Shannon RJ, Runswick MJ, Walker JE, Hirst J: **GRIM-19, a cell death regulatory gene product, is a subunit of bovine mitochondrial NADH:ubiquinone oxidoreductase (complex I)**. *J Biol Chem* 2001, **276**(42):38345--38348.

11. Rual J-Fo, Venkatesan K, Hao T, Hirozane-Kishikawa T, Dricot Al, Li N, Berriz GF, Gibbons FD, Dreze M, Ayivi-Guedehoussou N *et al*: **Towards a proteome-scale map of the human protein-protein interaction network**. *Nature* 2005, **437**(7062):1173--1178.

12. Murray J, Taylor SW, Zhang B, Ghosh SS, Capaldi RA: **Oxidative Damage to Mitochondrial Complex I Due to Peroxynitrite**. *J Biol Chem* 2003, **278**(39):37223-37230.

13. Mishmar D, Ruiz-Pesini E, Mondragon-Palomino M, Procaccio V, Gaut B, Wallace DC: **Adaptive selection of mitochondrial complex I subunits during primate radiation**. *Gene* 2006, **378**:11--18.

14. Nouws J, Nijtmans L, Houten SM, van den Brand M, Huynen M, Venselaar H, Hoefs S, Gloerich J, Kronick J, Hutchin T *et al*: **Acyl-CoA dehydrogenase 9 is required for the biogenesis of oxidative phosphorylation complex I**. *Cell Metab* 2010, **12**(3):283-294.

15. Carilla-Latorre S, Gallardo ME, Annesley SJ, Calvo-Garrido J, Grana O, Accari SL, Smith PK, Valencia A, Garesse R, Fisher PR *et al*: **MidA is a putative methyltransferase that is required for mitochondrial complex I function**. *J Cell Sci* 2010, **123**(10):1674-1683.

16. Szklarczyk D, Franceschini A, Kuhn M, Simonovic M, Roth A, Minguez P, Doerks T, Stark M, Muller J, Bork P *et al*: **The STRING database in 2011: functional interaction networks of proteins, globally integrated and scored**. *Nucleic Acids Res* 2011, **39**(Database issue):D561-568.

17. Saada A, Edvardson S, Rapoport M, Shaag A, Amry K, Miller C, Lorberboum-Galski H, Elpeleg O: **C6ORF66 is an assembly factor of mitochondrial complex I**. *Am J Hum Genet* 2008, **82**(1):32-38.
